# Stage-Specific Processing in Numerosity Working Memory: ERP Evidence for Load and Mismatch Effects in a Delayed Match-to-Sample Task

**DOI:** 10.3390/neurosci7020039

**Published:** 2026-03-20

**Authors:** Mengyu Duan, Zhuorui Liu, Li Sui

**Affiliations:** School of Health Science and Engineering, University of Shanghai for Science and Technology, Shanghai 200093, China; 233352499@st.usst.edu.cn (M.D.);

**Keywords:** numerosity, working memory, delayed match-to-sample, event-related potentials (ERP), mismatch effect, numerosity load, cognitive control

## Abstract

Numerosity can be represented in symbolic formats and non-symbolic dot arrays. How numerosity load unfolds across WM encoding/maintenance and test-stage comparison within a single paradigm remains unclear, especially within the tested 4–6 range. We used a delayed match-to-sample task manipulating numerosity (4–6) and match status, with two test blocks (dot–digit and dot–dot). Behaviorally, a higher numerosity reduced accuracy and increased RTs in both blocks, with larger costs in dot–dot; the mismatch reliably slowed RTs. At sample onset, occipital P1 and N1 amplitudes decreased with increasing numerosity, consistent with greater perceptual/processing demands at higher load, with the strongest differences at the high end of the range. During the delay, numerosity modulation was temporally specific, emerging in the 450–650 ms posterior window and remaining significant after FDR correction across the four consecutive delay windows. At the test, the mismatch elicited a more negative N2 in both blocks (larger in dot–dot), while numerosity also modulated N2 only in dot–dot, showing a monotonic increase in negativity with load. Controlling for condition-mean logRT did not eliminate these N2 effects. P3 showed no reliable modulation, whereas a later positive component was enhanced by mismatch selectively in dot–dot. Together, these results indicate stage-differentiated effects: numerosity load impacts early encoding and a circumscribed maintenance interval, whereas mismatch effects arise primarily during the test-stage comparison, with additional late evaluative activity when formats are aligned.

## 1. Introduction

Humans process numerical information from both symbolic formats (e.g., Arabic digits) and non-symbolic formats (e.g., dot arrays). These two formats are thought to rely on partially distinct representational levels, with symbolic numbers being more strongly linked to abstract semantic codes and non-symbolic numerosities being more strongly constrained by perceptual magnitude cues [1,2,3]. Electrophysiological evidence suggests that the brain can be sensitive to numerosity remarkably early: numerosity-related neural sensitivity has been reported as early as ~75 ms after visual input, and numerosity-dependent modulations have also been observed around ~180 ms, implying rapid processing of numerical information along the visual stream [4,5]. Converging with this view, other work has localized such early effects to stages associated with early visual cortex, showing that the responses around ~90 ms may already discriminate dot-array numerosity, thereby challenging the accounts that treat numerosity processing is purely dependent on later, high-level abstraction [6]. Nevertheless, whether numerosity is automatically extracted at early latencies across stimulus contexts—and how stable these early effects are across experimental settings—remains debated. A prevailing explanation is that dot-array numerosity can co-vary with continuous visual properties (e.g., density and total area), and that task goals and evidence-utilization strategies may differentially weight these cues, yielding heterogeneous timing and robustness of numerosity effects across paradigms [7,8,9].

Within the ERP literature, visual shape adaptation effects can emerge in early components, whereas numerosity adaptation effects often appear later, highlighting the extent to which task demands and stimulus structure can shape the temporal profile of the numerical effects [10]. In addition, numerosity processing has long been discussed in terms of whether small-number and large-number mechanisms are separable, yet ERP evidence for numerosities beyond 4 remains comparatively sparse and calls for further examination under stricter task control [2,5,11]. Accordingly, the present study focuses on numerosities 4–6, a moderate range near the classic boundary at four, to reduce potential confounds arising from strategy and mechanism differences associated with very small subitizing ranges versus very large approximate estimation ranges. Moreover, we aimed to decompose numerosity effects into load-related trends and endpoint contrasts at the high-load end, enabling a more precise characterization of continuity and potential boundary-like features within the 4–6 interval.

Placing numerosity processing within a working memory (WM) framework further raises fundamental questions about how numerical information is maintained and when it is recruited for decisions. The delayed match-to-sample (DMTS) task is a classic paradigm for WM research because its structure distinguishes encoding and maintenance during the sample period from comparison and decision processes during the test period, thereby enabling stage-specific inferences about the underlying cognitive and neural mechanisms [12]. A Brain Map meta-analysis indicates that successful DMTS performance recruits a distributed fronto-parietal network, and it also implies that without stage-anchored electrophysiological measures, the comparison and response-selection processes can easily contaminate inferences about maintenance mechanisms [12]. Consistent with this need for stage-resolved analysis, the fMRI decoding work on visual numerosity WM has emphasized that how and where numerosity is encoded during the delay period remains unclear and may involve representational transformations across maintenance; these findings further implicate intraparietal sulcus-related regions in numerosity representation [13]. In ERP research, posterior slow waves and the contralateral delay activity (CDA) have been repeatedly used to index resource demands and representational stability during maintenance, providing an operational electrophysiological basis for separating encoding/maintenance from comparison/decision in time [14,15]. Thus, characterizing both stages within a unified paradigm and anchoring effects to their corresponding time windows may offer more testable inferences about how numerosity representations are maintained and how they constrain subsequent comparison processes.

In DMTS, the test phase is not merely a retrieval operation; rather, it requires a comparison between the external probe and the internally maintained representation. When probe and memory representations mismatch, the participants must rapidly engage mismatch detection, control adjustments, and ultimately decision confirmation. Importantly, a mismatch first manifests as a representational discrepancy signal at the comparison stage; whether it additionally recruits conflict–control operations needs to be evaluated with respect to component time windows and scalp topographies. The fronto-central N2 is widely considered to be sensitive to mismatch events while also being linked to more general cognitive control demands. Therefore, the functional interpretations of N2 in numerosity DMTS should be grounded in task structure and the level at which conflict arises rather than relying on component labels alone. A comprehensive review further emphasizes the functional heterogeneity of anterior N2, showing that it can index mismatch detection in some contexts and control recruitment in others, reinforcing the need to interpret N2 effects in relation to comparison requirements and evidence use [16].

Classic Stroop paradigms have been extensively used to investigate cognitive control because conflicts between stimulus input and goal-relevant representations reliably elicit detection and resolution processes. ERP evidence frequently associates interference with a negative component often labeled N450 (~300–550 ms), followed by a later positive component, LPC (~600–900 ms), which has been linked to later evaluative, confirmatory, or resolution-related processing [17,18,19]. Crucially, conflict is not reducible to generic difficulty; it also depends on the level at which conflict arises. In numerical Stroop work combined with EMG measures, N450 can remain relatively stable even when response conflict varies, suggesting that N450 may reflect stimulus-level or more abstract conflict processes rather than simple response competition [20]. Moreover, studies manipulating stimulus onset asynchrony (SOA) to dissociate temporal stages of conflict processing suggest that earlier negative deflections are more closely tied to conflict detection than conflict resolution, whereas LPC may reflect subsequent resolution and/or selection-related processing [21]. Taken together, these findings imply that when mismatch effects are observed during the DMTS test phase, the key question is not only whether conflict is present, but also which level of conflict is engaged and which ERP components reflect the corresponding processing stages. Accordingly, we anchored test phase mismatch effects to both an earlier index of detection/control initiation and a later index of evaluative/confirmatory processing to assess whether mismatch remains an early discrepancy signal or cascades into later-stage evaluation and confirmation.

Beyond external conflict, the content held in the WM can pre-activate representations and thereby reshape the temporal dynamics of conflict processing. In WM–Stroop paradigms, incongruent conditions have been reported to elicit stronger N2 and P3 activity during external stimulus processing, suggesting that the WM content can modulate both conflict detection and subsequent resolution-related operations [22]. Other work proposes that, relative to classic Stroop, conflict–control cascades may initiate earlier when conflict stems from an internally maintained representation, consistent with the view that internal maintenance and external attentional processing share representational substrates and control resources [22,23]. This line of evidence suggests that internal representations are not passively maintained but can rapidly participate in probe processing and trigger a cascade spanning early detection and later evaluative operations.

When the WM content is numerical, mismatch in DMTS essentially reflects a discrepancy between the external probe and the internal numerosity representation. Such mismatch may engage fronto-central detection/control indices (e.g., N2) and may also extend to later evaluative/confirmatory components (e.g., LPC). However, direct evidence remains limited for simultaneously addressing, within a single task framework, several key questions. First, do numerosity load effects and mismatch effects overlap within the same processing stage, or do they primarily manifest in different stages—sample (encoding/maintenance) versus test (comparison/decision)? Second, does test phase mismatch more closely resemble N2-linked detection/control initiation, or does it align more strongly with later conflict-related and evaluative signatures commonly discussed in numerical interference research? Third, in numerosities beyond 4, do load effects exhibit non-linear patterns, and does such load dependence modulate the magnitude and timing of mismatch effects? Finally, under cross-format comparison conditions, symbolic digits and non-symbolic numerosities may not be fully isomorphic at the representational level and in evidence extraction, implying that identical mismatch manipulations may not necessarily produce homologous neural effects in the same temporal windows. This motivates an integrated framework that simultaneously considers stage-resolved processing and format-dependent representation [1,3].

To address these issues, we employed a numerosity DMTS paradigm that jointly manipulated numerosity load and match status. Numerosity load included four, five, and six items, and the match status included match and mismatch trials; the analyses were strictly anchored to the sample and test stages. Although both symbolic and non-symbolic numerical formats were involved, our primary goal was to test—within a unified stage-based framework—whether load effects and mismatch effects can be mechanistically dissociated in time, thereby clarifying the relationship among numerosity representation, WM maintenance, and conflict-related processing. Furthermore, we incorporated trend analyses and planned endpoint contrasts for numerosity to distinguish linear accumulation components from potential boundary-like effects at the high-load end, enabling theory-driven inference within the 4–6 range.

Based on prior evidence, we hypothesized that early encoding-related components and maintenance-related slow-wave indices during the sample stage would be sensitive to numerosity load, showing systematic modulation as load increases from 4 to 6. For the test stage, we predicted that mismatch (relative to match) would elicit a larger fronto-central N2 and would be accompanied by differences in later positive activity associated with evaluative/confirmatory processing. Critically, we expected a stage-specific pattern whereby numerosity load effects would primarily emerge during the sample stage, whereas mismatch effects would primarily emerge during the test stage, with no stable interaction pattern. Finally, considering representational format differences, we further predicted that dot–dot matching would yield stronger mismatch-detection signatures than dot–digit matching and would be more likely to show evaluative/confirmatory differences at later stages.

## 2. Materials and Methods

### 2.1. Participants

We recruited 32 undergraduate students (16 males and 16 females; age range: 19–23 years, M ≈ 20.1, SD ≈ 1.1). All participants were right-handed, had normal or corrected-to-normal vision, and reported no history of neurological or psychiatric disorders, recent substance abuse, or severe sleep problems. All participants volunteered to take part in the study and provided written informed consent prior to the experiment. They received course credit after completion.

For EEG analyses, four participants were excluded due to excessive artifacts (i.e., >40% of trials rejected in some conditions), which resulted in a substantial reduction and severe imbalance of usable trials across conditions, compromising the reliability of ERP averages and statistical inference. Thus, the final EEG sample included 28 participants (13 females; age range: 19–23 years). Behavioral analyses were conducted on the full sample of 32 participants.

### 2.2. Design and Materials

We employed a DMTS paradigm adapted from prior DMTS work on numerosity processing in working memory [24,25,26]. Each participant completed two task blocks: a dots-to-digit matching block (dot–digit) and a dots-to-dots matching block (dot–dot). The trials were randomized within each block. A mandatory break of at least 3 min was provided between blocks to reduce fatigue. Each participant completed 896 trials in total (448 trials per block).

Within each block, the core numerosity levels were 4, 5, and 6. Each numerosity contributed 128 trials, and match/mismatch trials were strictly balanced (64 trials each). To discourage strategic responding based on extreme numerosities, we included 3 and 7 as filler levels (64 trials per block in total; 32 trials each, with match/mismatch balanced). Only the trials with sample numerosity 4/5/6 were entered into the formal statistical analyses; the trials with sample numerosity 3 or 7 served solely to increase uncertainty.

In the dot–digit block, the sample stimulus was a dot array and the test stimulus was an Arabic digit; the participants judged whether the digit’s numerosity matched the sample numerosity. In the dot–dot block, both the sample and test stimuli were dot arrays; the participants judged whether the two arrays contained the same number of dots. In match trials, sample and test numerosities were identical. In mismatch trials, the numerical distance between sample and test was fixed at 1 (e.g., sample = 4, test = 3 or 5). For mismatch trials with sample numerosities 4/5/6, adjacent test numerosities could include 3 or 7 when applicable, ensuring a numerical difference constant of 1.

The stimuli consisted of dot arrays and Arabic digits. Dot arrays were composed of black filled dots presented centrally on a gray background, with an approximately circular overall configuration. The dot size was jittered within a small range. Across numerosity conditions, we attempted to minimize reliance on low-level continuous cues (e.g., cumulative area, density, and spatial distribution) by keeping these properties as comparable as possible [26]. Dot numerosities ranged from 3 to 7, and multiple exemplars were generated for each numerosity and randomly presented during the experiment. Arabic digits (“3”–“7”) were displayed in black at the center of the screen, with font size adjusted so that the digits were roughly comparable in salience to the dot arrays.

Response mapping was identical across tasks: the participants pressed the “F” key for match and the “J” key for mismatch. The mapping and response hands were kept constant across the participants. The participants were instructed to respond as quickly and as accurately as possible.

### 2.3. Procedure

The experiment was conducted individually in a quiet, moderately lit, electrically shielded laboratory room. The stimuli were presented on an LCD monitor (refresh rate: 60 Hz; resolution: 1920 × 1080) positioned approximately 65–75 cm from the participant. The responses were collected via a standard keyboard. The experimental stimuli were created and administered using the E-Prime 3.0 software (Psychology Software Tools, Sharpsburg, MD, USA).

**Figure 1:**
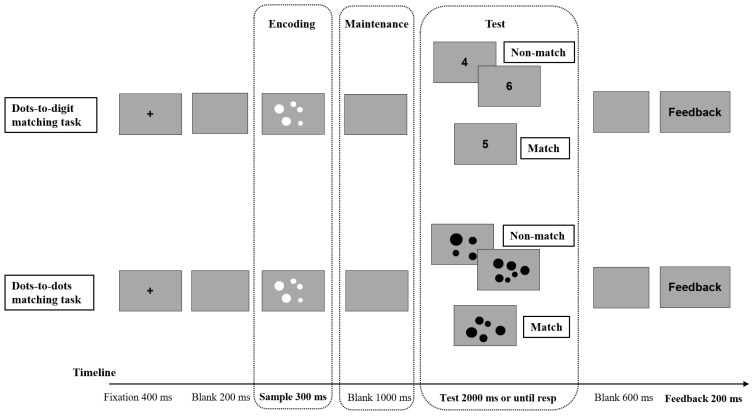
The depiction of the match-to-sample paradigm. In both tasks, the participants decided whether the numerosity of the test stimulus matched the numerosity encoded from the sample (match vs. non-match). In the dots-to-digit condition (**top row**), the test stimulus was an Arabic digit, whereas in the dots-to-dots condition (**bottom row**), the test stimulus was a dot array. The grey rectangles indicate the displays with a grey background used throughout the task, the plus sign indicates the fixation cross, and the dot arrays indicate the numerosity stimuli presented during the sample or test phase.

Before the formal experiment, the participants read on-screen instructions and completed practice trials for each task block to ensure that they understood the match/mismatch rules and key mapping. Additional practice was provided if performance was unstable, until the participants demonstrated adequate task understanding. The trial sequence is illustrated in Figure 1. Each trial began with a central fixation cross (“+”) for 400 ms, followed by a blank screen for 200 ms. The sample dot array was then presented for 300 ms. The participants were instructed to attend to the sample and memorize its numerosity without making any response. After sample offset, a blank delay interval lasted 1000 ms, during which participants maintained the numerosity in working memory. Next, the test stimulus appeared (an Arabic digit in the dot–digit block; a dot array in the dot–dot block) and remained on the screen until response or for a maximum of 2000 ms. The participants judged whether sample and test numerosities matched and responded using the assigned keys. After the response, a blank screen was shown for 600 ms, followed by brief response feedback for 200 ms, and then the next trial began.

### 2.4. Behavioral Data Analysis

The behavioral responses were recorded automatically in E-Prime. The trials with reaction times (RTs) shorter than 200 ms or longer than 2000 ms were excluded. Accuracy was computed over the remaining valid trials. The RT analyses were restricted to correct responses that also met the RT screening criterion.

Given the distributional characteristics of the behavioral measures, mixed-effects models were fitted separately for the dot–digit and dot–dot blocks. Accuracy was analyzed using generalized linear mixed-effects models with a binomial error structure and a logit link function. RTs were log-transformed prior to the analysis and analyzed using linear mixed-effects models. Match (match vs. mismatch) and numerosity were entered as fixed effects; numerosity was treated as an ordered predictor with centered coding (4/5/6 mapped to −1/0/1) to test monotonic load-related variation. A participant was included as a random intercept. All statistical analyses were conducted using IBM SPSS Statistics 26.0 (IBM Corp., Armonk, NY, USA), and all tests used a two-sided α level of 0.05.

### 2.5. EEG Recording and Analysis

#### 2.5.1. EEG Recording

Continuous EEG recordings were obtained throughout the test phase with a 32-channel EEG cap (Compumedics Neuroscan, Charlotte, NC, USA). The signals were sampled at 1024 Hz from 32 Ag/AgCl sintered electrode sites placed according to the international extended 10–20 system. An online reference was implemented using a cap-mounted reference electrode (REF) positioned on the midline between Cz and Pz, and the ground electrode (GND) was placed at the frontal midline site. Vertical and horizontal EOG were recorded using electrodes placed below the right eye and at the outer canthus of the right eye, respectively, to aid in identifying and correcting ocular artifacts. Electrode impedances were kept below 5 kΩ throughout the recording.

#### 2.5.2. Preprocessing

The preprocessing of the EEG signals was conducted using Curry 8 (Compumedics Neuroscan, Charlotte, NC, USA). The continuous data were band-pass filtered between 0.1 and 30 Hz using an FIR filter, and a notch filter was applied in the 48–52 Hz range to attenuate line noise. The data were then down-sampled to 512 Hz and re-referenced to the common average. After removing segments with prominent movement or muscle artifacts, we performed independent component analysis (ICA) and removed components associated with blinks and eye movements to minimize ocular contamination.

The data were epoched time-locked to sample onset and test onset. Sample-locked epochs spanned from −200 ms to 1300 ms, and test-locked epochs spanned from −200 ms to 800 ms. Baseline correction was applied using the −200 to 0 ms interval for both epoch types. The trials with voltages exceeding ±75 μV at any electrode were rejected. For each participant, ERPs were obtained by averaging artifact-free epochs for each condition. Sample-locked averages included all artifact-free trials, whereas test-locked averages included only correct trials that survived artifact rejection [27].

#### 2.5.3. ERP Measures and Statistical Analyses

Guided by prior ERP studies on numerosity processing and visual working memory maintenance that commonly define time windows and scalp topographies and informed by the present grand average waveforms and topographic maps to identify the most stable temporal intervals and regions, we partitioned the ERP analyses by task stage into two components—sample presentation/delay maintenance and test-locked comparison/decision—to characterize stage-specific processing [14,25,27].

Sample-locked measures. P1 (80–130 ms) and N1 (130–200 ms) mean amplitudes were extracted at occipital sites (O1/Oz/O2). A fronto-central N2 (220–320 ms) was quantified over F3/Fz/F4, FC3/FCz/FC4, and C3/Cz/C4. Sustained posterior activity was extracted from posterior ROIs (P7/P3/Pz/P4/P8, CP3/CP4, O1/Oz/O2) across four consecutive windows (300–450, 450–650, 650–900, and 900–1200 ms). The effects of numerosity were tested with within-subject RM-ANOVAs across Num (4/5/6) with Greenhouse–Geisser correction when warranted; polynomial contrasts (linear, quadratic) were used to characterize ordered numerosity trends. To control the multiple testing risk across the four posterior delay windows, *p*-values for the numerosity effect (Greenhouse–Geisser-corrected) were corrected using the Benjamini–Hochberg FDR procedure.

Test-locked measures. The analyses were conducted separately for the dot–digit and dot–dot blocks. N2 (220–320 ms) was quantified over F3/Fz/F4, FC3/FCz/FC4, and C3/Cz/C4; P3 (300–500 ms) and a late positive component (450–650 ms) were quantified over CP3/CP4 and P3/Pz/P4. Mean amplitudes were first analyzed using 2 (match: match vs. mismatch) × 3 (Num: 4/5/6) RM-ANOVAs with Greenhouse–Geisser correction and polynomial contrasts.

Additional control analyses. To evaluate whether N2 modulations could be explained by decision difficulty, we fitted mixed-effects models for N2 within each block, treating numerosity as a continuous predictor (−1/0/1 for 4/5/6) and including condition-mean logRT as a covariate, with participant modeled as a random intercept.

## 3. Results

### 3.1. Behavioral Results

#### 3.1.1. Dot–Digit Block

Accuracy: Accuracy showed a small but reliable effect of match, F(1, 12,035) = 6.493, *p* = 0.011 (OR = 1.253, 95% CI [1.053, 1.491]; higher on mismatch). Numerosity also exerted a robust monotonic effect, F(1, 12,035) = 42.937, *p* < 0.001 (OR = 0.725, 95% CI [0.629, 0.836] per one-step increase in numerosity). The match × numerosity interaction was not significant, F(1, 12,035) = 0.364, *p* = 0.546. Descriptively, accuracy declined across numerosity levels (Num 4: M = 0.961; Num 5: M = 0.949; Num 6: M = 0.929).

Reaction times: Log-transformed RTs (correct trials) were slower on mismatch than match trials, F(1, 11,404.116) = 542.418, *p* < 0.001. RTs also increased with numerosity, F(1, 11,404.026) = 24.733, *p* < 0.001. The match × numerosity interaction was significant, F(1, 11,404.039) = 12.488, *p* < 0.001, indicating that numerosity-related slowing differed between match and mismatch trials.

#### 3.1.2. Dot–Dot Block

Accuracy: Numerosity showed a strong monotonic effect, F(1, 12,008) = 132.996, *p* < 0.001 (OR = 0.693, 95% CI [0.618, 0.776] per one-step increase). The main effect of match was not significant, F(1, 12,008) = 0.011, *p* = 0.916 (OR = 0.993, 95% CI [0.869, 1.134]). However, the match × numerosity interaction was significant, F(1, 12,008) = 7.145, *p* = 0.008 (interaction OR = 0.802, 95% CI [0.682, 0.943]). Descriptively, accuracy decreased with numerosity (Num 4: M = 0.935; Num 5: M = 0.918; Num 6: M = 0.854).

**Figure 2:**
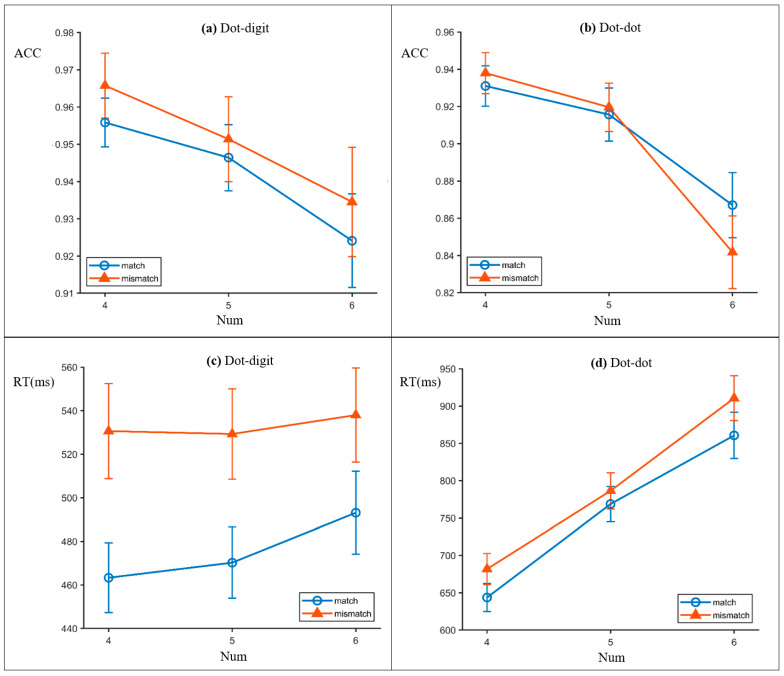
Behavioral performance in the delayed match-to-sample task. Mean accuracy and reaction time as a function of numerosity and match condition in the dots-to-digit block and the dots-to-dots block. The error bars indicate the standard error of the mean. (**a**) The accuracy in the dots-to-digit block. (**b**) The accuracy in the dots-to-dots block. (**c**) The reaction time in the dots-to-digit block. (**d**) The reaction time in the dots-to-dots block.

Reaction times: Log-transformed RTs (correct trials) were slower on mismatch than match trials, F(1, 10,872.138) = 76.669, *p* < 0.001. Numerosity exerted a very strong effect on RT, F(1, 10,872.173) = 1867.223, *p* < 0.001, with progressively slower responses as numerosity increased. The match × numerosity interaction was not significant, F(1, 10,872.072) = 0.180, *p* = 0.672. The overall behavioral pattern is illustrated in Figure 2.

### 3.2. EEG Results

#### 3.2.1. Sample-Locked ERP Results

**Figure 3:**
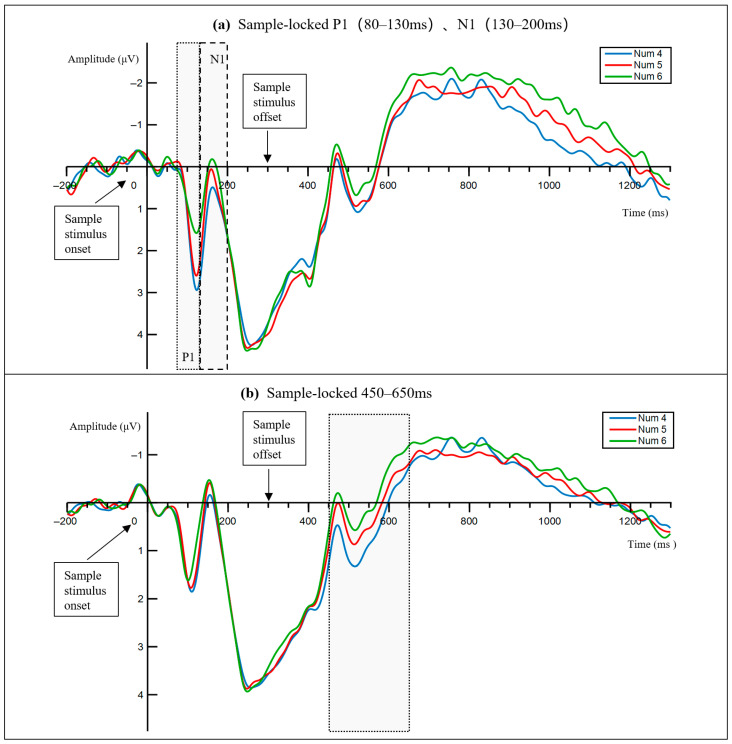
Sample-locked ERP waveforms illustrating the numerosity effects during the early encoding and late maintenance window. Grand average ERPs are shown for Num 4, Num 5, and Num 6. The shaded windows indicate the P1 interval, 80–130 ms, the N1 interval 130–200 ms, and the late delay activity interval 450–650 ms. (**a**) The sample-locked ERP waveform from the occipital ROI, highlighting P1 and N1. (**b**) The sample-locked ERP waveform from the posterior ROI, highlighting the 450–650 ms late delay activity window. The negative is plotted upward.

**Figure 4:**
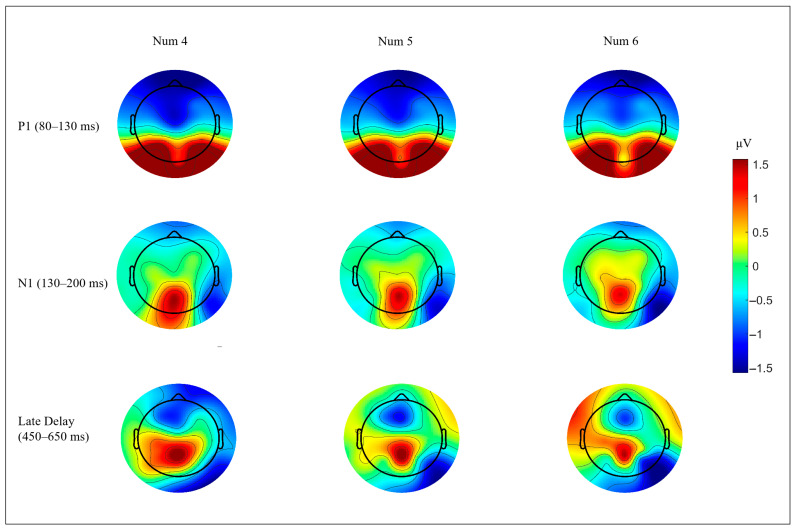
Scalp topographies of sample-locked activity across the numerosity levels. The topographical maps depict mean amplitude distributions for Num 4, Num 5, and Num 6 in three time windows. (**Top row**): P1 (80–130 ms). (**Middle row**): N1 (130–200 ms). (**Bottom row**): late delay activity (450–650 ms). The columns correspond to Num 4, Num 5, and Num 6, respectively. The color bars indicate amplitude in microvolts.

**Figure 5:**
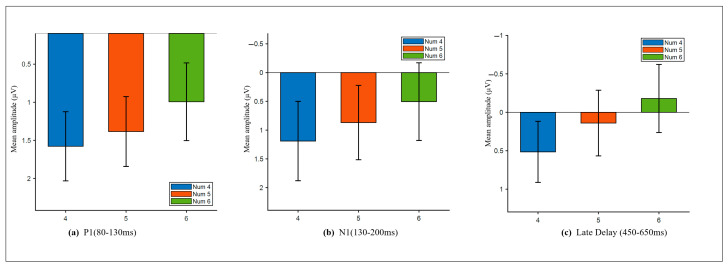
The mean amplitudes for sample-locked components and the late delay activity. The bars represent the mean amplitude and error bars indicate the standard error of the mean; individual participant values are overlaid as points. (**a**) Mean P1 amplitude (80–130 ms) at the occipital ROI for Num 4, Num 5, and Num 6. (**b**) Mean N1 amplitude (130–200 ms) at the occipital ROI for Num 4, Num 5, and Num 6. (**c**) Mean amplitude of the late delay activity window (450–650 ms) at the posterior ROI for Num 4, Num 5, and Num 6.

At occipital sites (O1, Oz, and O2), early visual activity varied with numerosity. P1 varied with numerosity, F(1.584, 42.765) = 8.05, *p* = 0.002, ηp^2^ = 0.230, and N1 showed a similar effect, F(1.478, 39.915) = 9.91, *p*= 0.001, ηp^2^ = 0.268. The trend analyses supported a linear decrease with increasing numerosity for P1, F(1, 27) = 10.875, *p*= 0.003, ηp^2^ = 0.288, and for N1, F(1, 27) = 12.409, *p* = 0.002, ηp^2^ = 0.315. Quadratic trends were not reliable (ps > 0.32). The occipital waveforms are shown in Figure 3a. Topographies for P1 and N1 are shown in Figure 4 (top and middle rows), and mean amplitudes are summarized in Figure 5a,b.

Fronto-central N2 activity in the 220–320 ms window, averaged over F3, Fz, F4, FC3, FCz, FC4, C3, Cz, and C4, did not show a significant numerosity effect (*p* = 0.105).

In the posterior ROI, numerosity effects were absent in the 300–450 ms, 650–900 ms, and 900–1200 ms windows (all ps > 0.18). In contrast, the 450–650 ms window showed a numerosity effect, F(1.243, 33.562) = 6.61, *p* = 0.010, ηp^2^ = 0.197, and a significant linear trend, F(1, 27) = 7.494, *p* = 0.011, ηp^2^ = 0.217. After Benjamini–Hochberg FDR correction across the four delay windows, only the 450–650 ms effect remained significant (q = 0.040). The posterior waveform is shown in Figure 3b. The corresponding topography is shown in Figure 4 (bottom row), and mean amplitudes are summarized in Figure 5c.

#### 3.2.2. Test-Locked ERP Results

**Figure 6:**
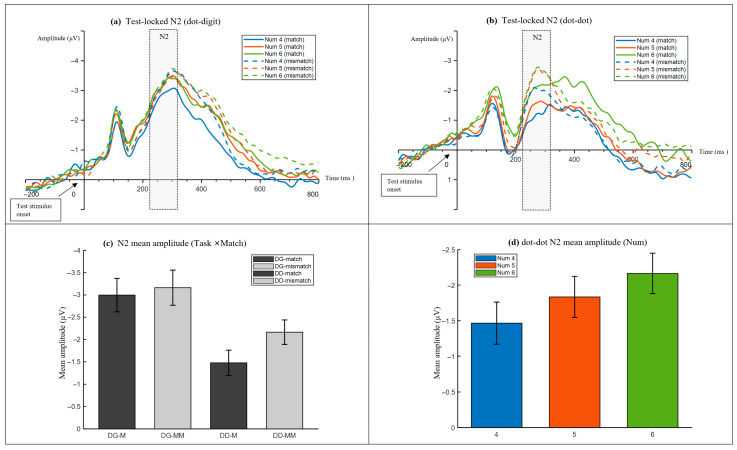
Test-locked N2 effects in the dots-to-digit and dots-to-dots blocks. Grand average ERPs are time-locked to test onset. The N2 analysis window (220–320 ms) is highlighted. The negative is plotted upward. (**a**) Test-locked N2 waveforms in the dots-to-digit block for Num 4/5/6 under match and mismatch conditions. (**b**) Test-locked N2 waveforms in the dots-to-dots block for Num 4/5/6 under match and mismatch conditions. (**c**) Mean N2 amplitude summarizing the match effect across task blocks (Task × Match). (**d**) Mean N2 amplitude showing the numerosity effect within the dots-to-dots block (Num 4/5/6).

In both task blocks, mismatch trials elicited a more negative N2 than match trials. In the dot–digit block, the main effect of match was significant, F(1, 27) = 5.246, *p* = 0.030, ηp^2^ = 0.163, with N2 being more negative for mismatch than match trials (match, M = −2.997 μV; mismatch, M = −3.163 μV). For the corresponding waveforms, see Figure 6a, and for the mean-amplitude summary, see Figure 6c. In the dot–dot block, the match effect was larger, F(1, 27) = 31.095, *p* < 0.001, ηp^2^ = 0.535, with N2 being more negative for mismatch than match trials (match, M = −1.477 μV; mismatch, M = −2.165 μV). For the corresponding waveforms, see Figure 6b, and for the mean-amplitude summary, see Figure 6c.

In the dot–dot block, N2 varied with numerosity, F(1.435, 38.747) = 7.963, *p* = 0.003, ηp^2^ = 0.228, becoming more negative as numerosity increased (Num 4 M = −1.465 μV, Num 5 M = −1.833 μV, and Num 6 M = −2.164 μV). The match by numerosity interaction was not significant in either block (all, *p* > 0.05). Polynomial contrasts confirmed a significant linear trend of numerosity for the dot–dot N2, F(1, 27) = 10.515, *p* = 0.003, ηp^2^ = 0.280. For the numerosity-related pattern, see Figure 6d.

Mixed-effects models were also fitted within each block with condition-mean logRT included as a covariate. In the dot–digit block, match was significant, F(1, 147.088) = 3.976, *p* = 0.048, and numerosity was significant, F(1, 137.370) = 4.120, *p* = 0.044, whereas logRT was not significant, F(1, 160.592) = 1.441, *p* = 0.232. In the dot–dot block, match was significant, F(1, 142.224) = 27.676, *p* < 0.001, and numerosity was significant, F(1, 162.987) = 4.689, *p* = 0.032, whereas logRT was not significant, F(1, 157.391) = 0.360, *p* = 0.549. Taken together, these results indicate that the N2 modulations were not fully explained by RT-related decision difficulty.

**Figure 7:**
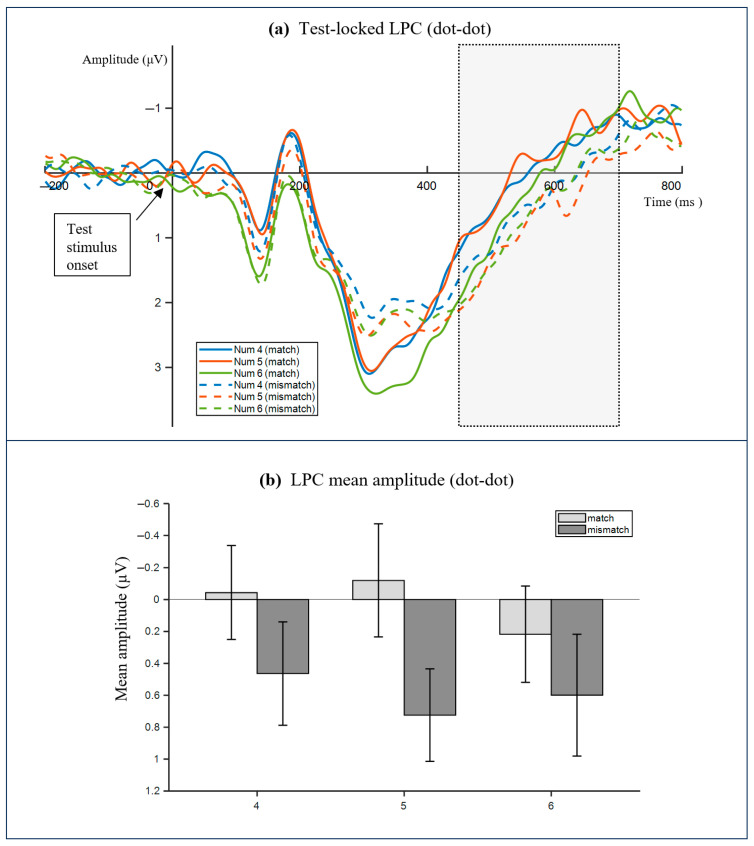
Test-locked late positive component in the dots-to-dots block. Grand average ERPs are time-locked to test onset for match and mismatch conditions. The LPC analysis window (450–650 ms) is highlighted. The negative is plotted upward. (**a**) Test-locked LPC waveforms in the dots-to-dots block for Num 4/5/6 under match and mismatch conditions. (**b**) Mean LPC amplitude (450–650 ms) in the dots-to-dots block for match and mismatch conditions; the error bars indicate the standard error of the mean.

P3 did not show reliable modulation by match, numerosity, or their interaction in either block (all, *p* > 0.05). For the late positive component in the 450–650 ms window, the effects differed across the blocks. In the dot–digit block, neither the main effect of match nor the match by numerosity interaction reached significance, and the main effect of numerosity was also not significant (all, *p* > 0.05). In the dot–dot block, the mismatch trials elicited a larger late positivity, yielding a significant main effect of match, F(1, 27) = 4.386, *p* = 0.046, ηp^2^ = 0.140, with mean amplitudes of −0.377 μV for match trials and 0.154 μV for mismatch trials. In this block, the main effect of numerosity and the match by numerosity interaction were not significant (all ps > 0.05). For the corresponding LPC waveforms and mean-amplitude summary, see Figure 7.

## 4. Discussion

In this study, we investigated how numerosity load and match status shape performance and neural dynamics in a delayed match-to-sample task, with analyses centered on sample-locked encoding/maintenance and test-locked comparison/decision processes. Across behavioral and ERP measures, numerosity and match status were dissociable in their temporal loci of influence, indicating that they do not operate in an isomorphic manner within the processing stream.

Numerosity load modulated early visual activity following sample presentation and was further expressed as increased resource demand within a circumscribed portion of the maintenance period. By contrast, match status exerted its clearest effects during the test phase, where mismatch trials elicited enhanced mismatch-related activity and, under within-format comparison, were followed by a later evaluative positivity. Consistent with this account, ordered trend tests and planned endpoint contrasts (Num 4 vs. Num 6) supported monotonic load-related variation while also revealing a reproducible high-load endpoint difference within the tested 4–6 range. However, given the restricted numerosity interval, these endpoint effects should be interpreted as within-range sensitivity rather than as evidence for a categorical boundary beyond the present design [28,29].

Behaviorally, both blocks exhibited increasing costs as numerosity increased, yet these costs were substantially larger in the dot–dot block: RTs rose steeply, with load and accuracy declining accordingly. This pattern is consistent with the view that, when comparison is performed within the same stimulus format, numerosity information more directly determines decision difficulty. In dot–digit judgments, RT costs were smaller and mismatch effects varied as a function of numerosity, plausibly reflecting additional mapping/alignment requirements in cross-format comparison [30]. Overall, the behavioral profile points to increased time demands during comparison/decision as the most stable manifestation of mismatch rather than a uniform degradation of the maintained representation [31,32].

At the sample-locked stage, occipital P1 and N1 varied with numerosity, with differences largely driven by Num 6 relative to Num 4/Num 5. Prior work has reported early sensitivity of visual ERPs to factors that co-vary with numerosity and perceptual load [4,6]. In the present design, because numerosity was sampled over a narrow interval (4–6) and low-level visual properties were not formally modeled as covariates, the P1/N1 effects are discussed conservatively as reflecting increased perceptual/processing demands at higher numerosity rather than unambiguous numerosity-specific encoding [7,8,9]. The ordered-trend pattern and the Num 4 vs. Num 6 contrast nonetheless indicate that the high-load condition already yields measurable differences during early processing, consistent with electrophysiological evidence from delayed numerosity matching paradigms [25].

No stable numerosity modulation was observed for the sample-locked fronto-central N2, suggesting that monitoring/control-related activity is not the primary locus of numerosity differences immediately following sample presentation. During maintenance, numerosity modulation was temporally specific: a reliable effect emerged only in the 450–650 ms interval and remained significant after Benjamini–Hochberg FDR correction across the four consecutive delay windows, whereas adjacent windows did not show comparable effects. This temporal localization argues against a sustained, uniform maintenance load and is more consistent with a stage-wise account in which higher numerosity imposes heightened resource pressure during a consolidation/stabilization phase after encoding [13,15,25].

At the test-locked stage, both blocks showed a more negative N2 for mismatch than match trials, with a substantially larger mismatch effect in dot–dot. Anterior N2 has been linked to mismatch-related processing and early control demands across tasks, albeit with known functional heterogeneity across contexts [16].

In the present paradigm, the match/mismatch relation between the test stimulus and the maintained representation is decision-diagnostic, making mismatch-related signaling a plausible interpretation of enhanced N2 negativity [16,33,34]. Numerosity additionally modulated test-locked N2 in dot–dot, with N2 becoming more negative as numerosity increased, suggesting that under within-format comparison, load can be incorporated early in the comparison-related processing cascade.

A central concern was whether these N2 modulations—particularly in dot–dot—could be accounted for by general decision difficulty. The control analyses incorporating condition-mean logRT as a covariate indicated that mismatch-related N2 effects remained significant in both blocks and that the numerosity effect in dot–dot persisted, whereas logRT itself was not significant. These results therefore indicate that the N2 modulations were not fully explained by RT-related decision difficulty, supporting a mismatch-related account while acknowledging that RT and N2 may partially co-vary [16,34].

Later components showed a different profile. P3 was not reliably modulated by match, numerosity, or their interaction in either block, suggesting that the primary dissociations were not expressed as classic updating-related activity [35]. By contrast, the late positive component diverged across blocks: dot–dot showed a mismatch-related LPC enhancement, whereas dot–digit did not. This pattern is compatible with a “detect-then-evaluate” sequence under within-format comparison, where stronger mismatch-related processing is followed by later evaluative confirmation. In cross-format dot–digit judgments, additional alignment between symbolic and non-symbolic representations may introduce strategy variability that attenuates late evaluative activity at the group level [2,30].

Given the restricted numerosity interval, these results are best interpreted in terms of load-dependent processing demands (and potentially strategy adjustments) rather than as definitive evidence for a categorical boundary or a general dissociation between small and large number mechanisms [5,36].

## 5. Conclusions

Using a delayed match-to-sample paradigm with dot–digit and dot–dot blocks, we observed a stage-differentiated pattern in which the numerosity load and match status influenced distinct processing phases. The numerosity load modulated early occipital activity during encoding and was expressed as a temporally specific maintenance-related effect in the delay period, whereas match status primarily affected test-locked comparison processes, eliciting enhanced mismatch-related N2 activity and—under within-format comparison—an additional late evaluative positivity. The control analyses incorporating condition-mean logRT indicated that the test-stage N2 effects were not fully attributable to RT-related decision difficulty. Although several effects were most pronounced at the high end of the tested range, these patterns are best interpreted as within-range sensitivity to increasing processing demands rather than evidence for a categorical boundary. Future work sampling a broader numerosity range and explicitly modeling low-level visual covariates will be essential for refining mechanistic interpretation.

## Data Availability

Data is unavailable due to privacy restrictions.

## Abbreviations

The following abbreviations are used in this manuscript.

ANOVA	analysis of variance
DMTS	delayed match-to-sample
ERP	event-related potentials
ROI	regions of interest
WM	working memory

## References

[1] Holloway I.D., Price G.R., Ansari D. (2010). Common and segregated neural pathways for the processing of symbolic and nonsymbolic numerical magnitude: An fMRI study. Neuroimage.

[2] Nieder A., Dehaene S. (2009). Representation of number in the brain. Annu. Rev. Neurosci..

[3] Eger E., Sterzer P., Russ M.O., Giraud A.L., Kleinschmidt A. (2003). A supramodal number representation in human intraparietal cortex. Neuron.

[4] Park J., DeWind N.K., Woldorff M.G., Brannon E.M. (2016). Rapid and Direct Encoding of Numerosity in the Visual Stream. Cereb. Cortex.

[5] Hyde D.C., Spelke E.S. (2009). All numbers are not equal: An electrophysiological investigation of small and large number representations. J. Cogn. Neurosci..

[6] Fornaciai M., Brannon E.M., Woldorff M.G., Park J. (2017). Numerosity processing in early visual cortex. Neuroimage.

[7] Gebuis T., Reynvoet B. (2012). The role of visual information in numerosity estimation. PLoS ONE.

[8] Leibovich T., Katzin N., Harel M., Henik A. (2017). From “sense of number” to “sense of magnitude”: The role of continuous magnitudes in numerical cognition. Behav. Brain Sci..

[9] Dakin S.C., Tibber M.S., Greenwood J.A., Kingdom F.A., Morgan M.J. (2011). A common visual metric for approximate number and density. Proc. Natl. Acad. Sci. USA.

[10] Soltész F., Szűcs D. (2014). Neural adaptation to non-symbolic number and visual shape: An electrophysiological study. Biol. Psychol..

[11] Sun J., Sun P. (2021). An ERP study of dissociated mechanisms for processing large and small numerosities. Stud. Psychol. Behav..

[12] Daniel T.A., Katz J.S., Robinson J.L. (2016). Delayed match-to-sample in working memory: A BrainMap meta-analysis. Biol. Psychol..

[13] Pennock I.M.L., Schmidt T.T., Zorbek D., Blankenburg F. (2021). Representation of visual numerosity information during working memory in humans: An fMRI decoding study. Human Brain Mapp..

[14] Luria R., Balaban H., Awh E., Vogel E.K. (2016). The contralateral delay activity as a neural measure of visual working memory. Neurosci. Biobehav. Rev..

[15] Vogel E.K., Machizawa M.G. (2004). Neural activity predicts individual differences in visual working memory capacity. Nature.

[16] Folstein J.R., Van Petten C. (2008). Influence of cognitive control and mismatch on the N2 component of the ERP: A review. Psychophysiology.

[17] Hanslmayr S., Pastötter B., Bäuml K.H., Gruber S., Wimber M., Klimesch W. (2008). The electrophysiological dynamics of interference during the Stroop task. J. Cogn. Neurosci..

[18] West R. (2003). Neural correlates of cognitive control and conflict detection in the Stroop and digit-location tasks. Neuropsychologia.

[19] Liotti M., Woldorff M.G., Perez R., Mayberg H.S. (2000). An ERP study of the temporal course of the Stroop color-word interference effect. Neuropsychologia.

[20] Szűcs D., Soltész F. (2012). Functional definition of the N450 event-related brain potential marker of conflict processing: A numerical Stroop study. BMC Neurosci..

[21] Appelbaum L.G., Boehler C.N., Won R., Davis L., Woldorff M.G. (2012). Strategic allocation of attention reduces temporally predictable stimulus conflict. J. Cogn. Neurosci..

[22] Wang W., Qi M., Gao H. (2021). An ERP investigation of the working memory stroop effect. Neuropsychologia.

[23] Kiyonaga A., Egner T. (2014). The working memory Stroop effect: When internal representations clash with external stimuli. Psychol. Sci..

[24] Sella F., Lanfranchi S., Zorzi M. (2013). Enumeration skills in Down syndrome. Res. Dev. Disabil..

[25] Fu W., Dolfi S., Decarli G., Spironelli C., Zorzi M. (2021). Electrophysiological Signatures of Numerosity Encoding in a Delayed Match-to-Sample Task. Front. Human Neurosci..

[26] Gebuis T., Reynvoet B. (2011). Generating nonsymbolic number stimuli. Behav. Res. Methods.

[27] Luck S.J. (2014). An Introduction to the Event-Related Potential Technique.

[28] Cowan N. (2001). The magical number 4 in short-term memory: A reconsideration of mental storage capacity. Behav. Brain Sci..

[29] Luck S.J., Vogel E.K. (1997). The capacity of visual working memory for features and conjunctions. Nature.

[30] Bulthé J., De Smedt B., Op de Beeck H.P. (2014). Format-dependent representations of symbolic and non-symbolic numbers in the human cortex as revealed by multi-voxel pattern analyses. Neuroimage.

[31] Larson M.J., Kaufman D.A., Perlstein W.M. (2009). Neural time course of conflict adaptation effects on the Stroop task. Neuropsychologia.

[32] MacLeod C.M. (1991). Half a century of research on the Stroop effect: An integrative review. Psychol. Bull..

[33] Donkers F.C., van Boxtel G.J. (2004). The N2 in go/no-go tasks reflects conflict monitoring not response inhibition. Brain Cogn..

[34] Botvinick M.M., Braver T.S., Barch D.M., Carter C.S., Cohen J.D. (2001). Conflict monitoring and cognitive control. Psychol. Rev..

[35] Polich J. (2007). Updating P300: An integrative theory of P3a and P3b. Clin. Neurophysiol..

[36] Hyde D.C., Spelke E.S. (2011). Neural signatures of number processing in human infants: Evidence for two core systems underlying numerical cognition. Dev. Sci..

